# The respective effect of under-rib convection and pressure drop of flow fields on the performance of PEM fuel cells

**DOI:** 10.1038/srep43447

**Published:** 2017-03-02

**Authors:** Chao Wang, Qinglei Zhang, Shuiyun Shen, Xiaohui Yan, Fengjuan Zhu, Xiaojing Cheng, Junliang Zhang

**Affiliations:** 1 Institute of Fuel Cell, School of Mechanical Engineering, Shanghai Jiao Tong University, Dongchuan Rd. 800, Shanghai, China

## Abstract

The flow field configuration plays an important role on the performance of proton exchange membrane fuel cells (PEMFCs). For instance, channel/rib width and total channel cross-sectional area determine the under-rib convection and pressure drop respectively, both of which directly influence the water removal, in turn affecting the oxygen supply and cathodic oxygen reduction reaction. In this study, effects of under-rib convection and pressure drop on cell performance are investigated experimentally and numerically by adjusting the channel/rib width and channel cross-sectional area of flow fields. The results show that the performance differences with various flow field configurations mainly derive from the oxygen transport resistance which is determined by the water accumulation degree, and the cell performance would benefit from the narrower channels and smaller cross sections. It reveals that at low current densities when water starts to accumulate in GDL at under-rib regions, the under-rib convection plays a more important role in water removal than pressure drop does; in contrast, at high current densities when water starts to accumulate in channels, the pressure drop dominates the water removal to facilitate the oxygen transport to the catalyst layer.

Flow field is a key component of proton exchange membrane fuel cells (PEMFCs). It not only dominates the delivery of the reactants and the removal of products, but also serves an important role in evenly distributing reactants over the entire catalyst layer (CL), which results in uniform current distributions, large discharging current and high power density^1^. A critical challenge for flow field design is how to improve the water management via a suitable flow field pattern^2^: firstly, the flow field should remove the accumulated water efficiently to avoid the flooding issue; secondly, flow field should enable a high performance with low accessory power loading^3^,^4^.

The high pressure drop can efficiently remove accumulated water in flow field, but increases the accessory power loading of a fuel cell stack. The ideal flow field is expected to overcome the flooding issue with low pressure drop. The typically used flow fields, *i.e.,* interdigitated, parallel and serpentine flow fields, result in different levels of pressure drop. Interdigitated flow field^5^,^6^,^7^ consists of ended channels, which forces the gases to flow into the gas diffusion layer (GDL), promoting water removal. Such a design shows a high performance at high current densities when water is produced significantly, but its pressure drop is also the highest among these three types of flow fields. Pressure drop within parallel flow field is low, but water is prone to accumulate in channels, resulting in water flooding^8^,^9^,^10^. The serpentine flow field is considered as a compromised design, which has higher pressure drop than parallel flow field due to the long channel length and numerous turnings. However, for serpentine flow field, water flooding can still occur at the outlet region^11^,^12^,^13^ and U-bend regions^14^; in addition, membrane dehydration might occur at the inlet region. Therefore, some work has been done for the optimization of serpentine flow field to keep a balance between the water removal and pressure drop. For instance, Belchor *et al*.^15^ developed a parallel serpentine-baffle flow field which used a parallel-serpentine design at gas inlet and an interdigitated one at the outlet. This flow field enabled a good performance at low humidification conditions. Suresh *et al*.^16^ developed a “parallel” serpentine flow field. Three split serpentine regions laid out parallelly in one flow field, which not only reduced the pressure drop, but also replenish oxygen in the oxygen-deficient portions of the serpentine channel.

Besides the pressure drop, the under-rib convection is another critical factor that influences water management^17^,^18^,^19^,^20^. It facilitates liquid water removal in land regions and increases the effective utilization of catalysts^21^,^22^,^23^. As the channel geometry, *i.e.,* width, depth and shape, directly correlates with the under-rib convection, efforts have been made on the channel geometry design to reveal the effects of under-rib convection and to improve the water management. Yoon *et al*.^24^ investigated the effect of channel area portion with the same channel width. They found that a higher channel area portion leaded to better performance. Scholta *et al*.^25^ found that narrower channels were beneficial for cell performance at high current densities, whereas wider ones were preferred at low current densities. Goebel^26^ studied the impact of land width and channel span on fuel cell performance with a land width ranging from 0.25 mm to 1 mm. At high current densities, the land width was found to be the dominant factor, and the 0.25/0.25 mm channel/rib showed the best performance. Inoue *et al*.^27^ systematically investigated the influence of channel depth on the current distribution. A shallow channel performed better than the deep one did, because the differential pressure between adjacent channels was increased with the shallow channels, enhancing the through-plane transport of oxygen in GDL. Channels with convergent depth also resulted in a better cell performance by forcing gas flowing into the gas diffuser layer and catalyst layer^28^,^29^,^30^,^31^,^32^.

Water removal in fuel cells is determined by both the pressure drop and under-rib convection. Although great efforts have been made to utilize the pressure drop and under-rib convection to improve water management, only the general relationship between these two behaviors and the cell performance is obtained as they are coupled with each other. To reveal the detailed mechanism, four parallel-serpentine flow fields with different channel geometries are designed and fabricated in this study. Both experimental and simulative methods are used to study the respective effects of under-rib convection and pressure drop on the cell performance via adjusting the operation conditions, the channel/rib width and the channel cross sectional area of flow channels. The experimental and simulative results demonstrate that at low current densities, when water mainly accumulates in GDL, the under-rib convection plays a more important role in water removal than pressure drop does. At high current densities, when a large amount of water accumulates in channels, the pressure drop dominates the water removal to facilitate the oxygen transportation.

## Results and Discussion

### Design of Flow Field Patterns

**Figure 1:**
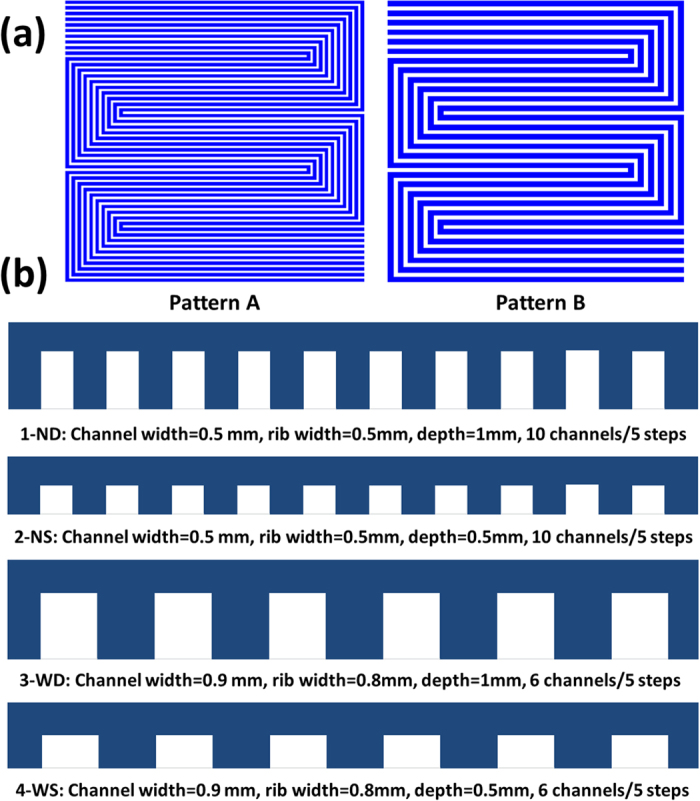
The patterns **(a)** and channel geometry **(b)** of flow fields designed in this study.

In this study, the under-rib convection was adjusted by channel/rib width and the pressure drop was adjusted by the total channel cross section area. Narrow channel/rib width facilitates the under-rib convection and small channel cross section results in high pressure drop. The four 25 cm^2^ (5 cm × 5 cm) flow fields with 5-steps were designed. Pattern A with narrow & deep channels (1-ND) and narrow & shallow channels (2-NS) were respectively fabricated which own 10-passes (Fig. 1a). Pattern B with wide & deep channels (3-WD) and wide & shallow channels (4-WS) were also fabricated with 6-passes. To enhance under-rib convection, the width of channels and ribs in Pattern A is designed to be as narrow as 0.5 mm (Fig. 1b). In Pattern B, the width of channels and ribs was widened to 0.9 mm and 0.8 mm respectively for comparison. The channel depth was adjusted to obtain various channel cross section area. 1-ND and 3-WD had deep channels of 1 mm; meanwhile, 2-NS and 4-WS had shallow channels of 0.5 mm. From 1-ND to 4-WS, the total cross sectional area of all channels was 5.0, 2.5, 8.5 and 4.2 mm^2^ respectively.

### Cell performance in Air

**Figure 2:**
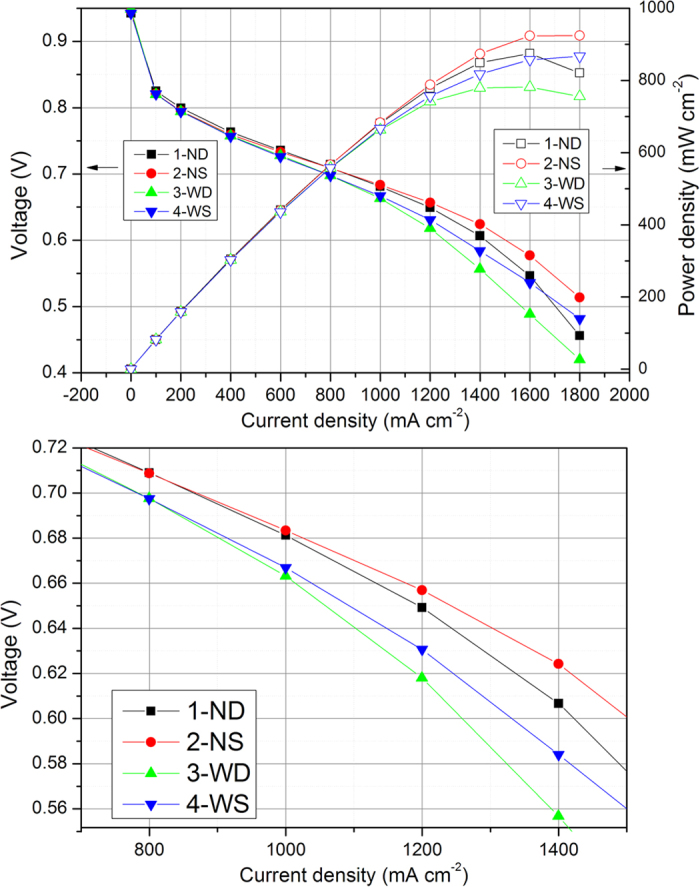
The cell performance with designed flow fields **(a)** and the performance highlighted from 800 to 1400 mA cm^−2^ **(b)** at 100% RH (Cell Temp. = 80 °C and Back Pressure = 150 kPa).

The performance with these flow field patterns were tested at H_2_/Air condition at 80 °C and 100% RH. As shown in Fig. 2(a), the IV curves can be divided into three regions to describe the performance difference among these fuel cells with various flow field patterns. The first part is from 0 mA cm^−2^ to 500 mA cm^−2^, where the performance is dominated by the activation polarization. It shows that there is no performance difference with different flow fields, because only a small amount of water is produced at low current densities which would not lead to the flooding issue. Thus, the effect of the flow fields is negligible at this operating condition.

The second region is from 500 mA cm^−2^ to 1100 mA cm^−2^. It is found that the cell performance is mainly influenced by the channel/rib width in this region. 1-ND and 2-NS with narrow design shows the same voltage of 0.710 V at 800 mA cm^−2^; in contrast, the voltage with 3-WD and 4-WS with wide channels/ribs is 0.697 V, which is approx. 10 mV lower. Two phenomena can be found that (1) the patterns with the same channel/rib width shows similar cell voltage, and (2) narrower designs enable better performance. Flooding firstly occurs in the GDL at the under-rib regions, where the accumulated water has to be removed by the under-rib gas convection. Thus, flow fields with the same channel/rib width are prone to show the same performance. Since the under-rib flow velocity of oxygen was be inversely proportional to the rib width^22^, 1-ND and 2-NS with narrow ribs would enhance the under-rib convection at 800 mA cm^−2^ to facilitate water removal in the GDL, in turn improving the oxygen transport to the catalyst layer and enabling a better performance than 3-WD and 4-WS do.

The third region ranges from 1100 mA cm^−2^ to 1800 mA cm^−2^. Different with the second region, the flow fields with the same channel-rib width yield an obvious performance difference which is highlighted in Fig. 2(b). In the third region, 1-ND and 3-WD with deep channel design shows sharper voltage decline than 2-NS and 4-WS does. At the high current densities, the liquid water accumulates not only in the GDL, but also in flow channels, which makes the pressure drop play a critical role in water removal. At 1100 mA cm^−2^, the voltage difference between 1-ND and 2-NS based fuel cells is only 5 mV, but the difference increases to 18 mV at 1400 mA cm^−2^. In the same current range, the voltage difference between 3-WD and 4-WS even increases from 13 mV to 26 mV. It tells that the shallow flow fields have higher pressure drop, which would facilitate the removal of water accumulated in channels especially at high current densities.

**Figure 3:**
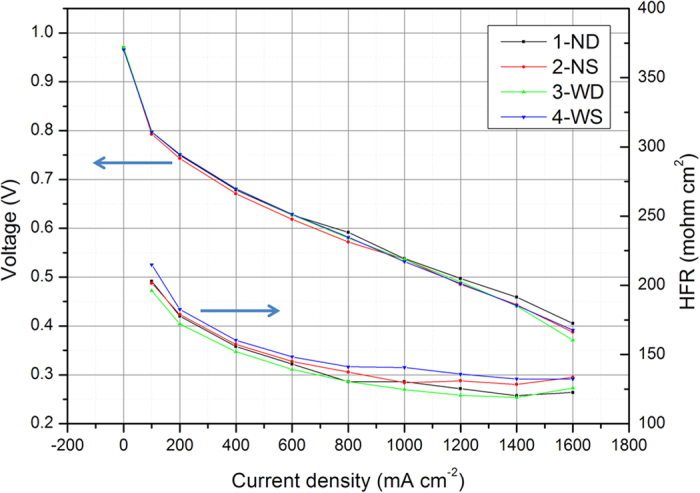
The cell performance and HFR measurement with designed flow fields at 37% RH (Cell Temp. = 80 °C and Back Pressure = 150 kPa).

To confirm the influence of different flow fields on water removal, the cell performance at low humidity was also tested at 37% RH. As shown in Fig. 3, fuel cells with different flow fields have almost the same performance and no sharp voltage decline appears at high current densities. This is because intense water flooding rarely occurs at such low humility condition and there is no concern about the oxygen transportation. Therefore, the channel/rib width, as well as the channel cross sectional area, will not exhibit a remarkable influence on the cell performance. Based on these experimental results, it can be concluded that the channel geometry of the parallel-serpentine flow fields we investigate in this study (channel/rib width & channel cross sectional area) dominates the inside water distribution and subsequently influences the oxygen transport in fuel cell.

### The EIS Analysis

**Figure 4:**
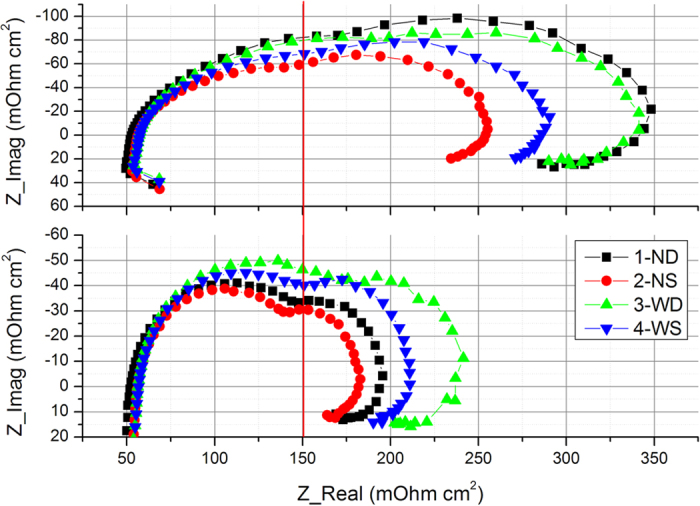
The EIS response with designed flow fields at 0.6 V and 0.7 V at 100% RH (Cell Temp. = 80 °C and Back Pressure = 150 kPa).

The EIS at 0.6 V and 0.7 V was conducted to investigate the *in-situ* state of the cell at RH of 100%. EIS results of the H_2_/Air cell show two semi arcs. The left arc at high frequency refers to the charge transfer resistance, and the right one at low frequency refers to the mass transport resistance (MTR). In Fig. 4, the left arcs (ca. lower than 150 mOhm cm^2^) do not change obviously as the voltage decreases from 0.7 V to 0.6 V, indicating that the effects of channel shapes on the charge transfer is negligible. However, there is a remarkable difference in the right arcs, which stands for mass transport resistance at different voltages. From 0.7 V to 0.6 V, radius of the low-frequency arc increases almost two times, indicating a much larger mass transport resistance. For clearness, the MTR results are also summarized in Table 1. At 0.7 V, the low-frequency impedance with 2-NS reaches 194 mOhm cm^2^, which increases to 255 mOhm cm^2^ at 0.6 V. This could be attributed to the increased oxygen transport resistance caused by the water accumulation, since more water is produced at 0.6 V by the intense cathodic oxygen reduction reaction.

With the increase in current density, these four flow fields show different states in the mass transport controlled arc. At 0.7 V, the mass transport resistances of flow fields with the same rib width are almost same. For the narrow channels, the width of MTR with 1-ND and 2-NS is 43 mOhm cm^2^ and 46 mOhm cm^2^ respectively. In contrast, 3-WD and 4-WS reaches to 65 mOhm cm^2^ and 62 mOhm cm^2^ respectively. The MTR with narrow channels is apparently smaller than that with the wide ones. This is because that less water is produced at 0.7 V and the water existing in channels can be easily removed. Thus, accumulated water in GDL at under-rib regions is the main reason hindering the oxygen transport. Narrow channels can enhance the under-rib convection to remove excess water in GDL, therefore resulting in a smaller MTR. However, the MTR becomes more relevant to the channel depth than to the rib width at 0.6 V. For instance, the MTR of 1-ND is 167 mOhm cm2, 50 mOhm cm2 higher than that of 2-NS.3-WD also has a higher MTR than 4-WS. At high current densities large amount of water accumulates in the channels. The water removal in channels is driven by the pressure drop, thus a shallower channel or a smaller total channel cross sectional area increases the pressure drop and results in low MTR.

The EIS results illustrate the cell performance shown in Fig. 2(b). At 0.7 V, the narrow channel patterns show better performance as they enable an enhanced under-rib convection to remove the accumulated water in GDL, and it can be denoted as “rib-controlled region”. At 0.6 V, water accumulation in channels becomes dominant; therefore, a shallow channel patterns is needed as it can remove waters in channels efficiently by the high pressure drop.

### The influence on mass transportation

**Figure 5:**
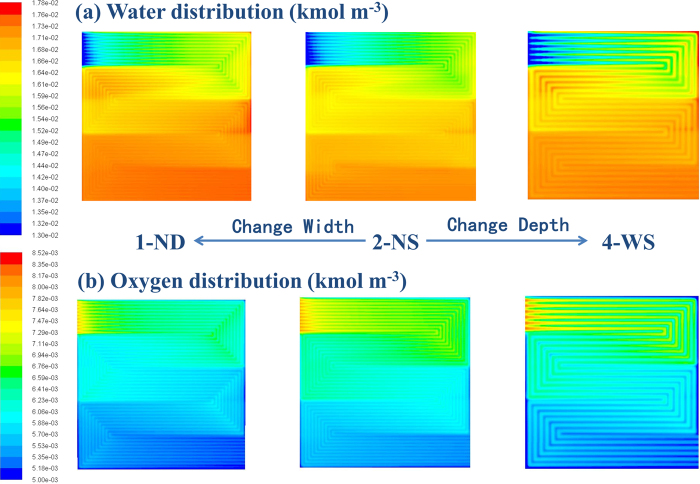
The water and oxygen concentration distribution in GDL with 1-ND, 2-NS and 4-WS at 1000 mA cm^−2^ at 100% RH (Cell Temp. = 80 °C and Back Pressure = 150 kPa).

Since the mass transportation is directly relevant to the flow field design, the water behavior in the GDL was studied by the computational fluid dynamics modeling at 1000 mA cm^−2^ (RH = 100%, Back Pressure = 150 kPa) with different flow fields. Figure 5(a) shows the water distribution with varying channel/rib width and depth. When the channel depth increases from 0.5 mm (2-NS) to 1.0 mm (1-ND), the pressure drop decreases; but there is no obvious change in the water distribution. However, when the channel/rib width changes from 0.5 mm (2-NS) to 0.9 mm (4-WS), water gradient under adjacent rib and channel becomes much larger. It reveals that the water distribution in GDL is mainly determined by the under-rib convection.

**Figure 6:**
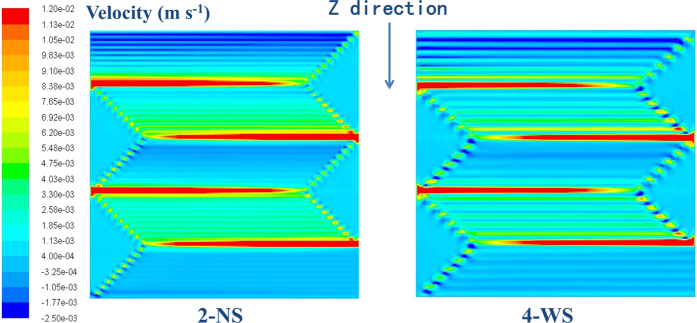
The flow velocity in the z direction (across rib) with 2-NS and 4-WS at 1000 mA cm^−2^ at 100% RH (Cell Temp. = 80 °C and Back Pressure = 150 kPa).

Figure 6 shows the flow velocity of air in GDL at z-direction (convection across the ribs) at 1000 mA cm^−2^. With a narrow rib width, 2-NS shows a higher velocity across ribs and more uniform velocity distribution than 4-WS does. The high z-direction flow velocity with 2-NS can promote water removal in GDL at under rib regions efficiently, which improves the oxygen transportation and the cell performance consequently. As shown in Fig. 5(b), the oxygen concentration gradient between under-rib and under-channel regions is much lower in 2-NS map than that in 4-WS, which indicates that narrow patterns are preferable for oxygen transport at under rib regions. The computational modeling is consistent with the experiment results. 2-NS and 1-ND with the same channel/rib width also show similar water distribution and performance at 1000 mA cm^−2^ (0.681 V for 1-ND and 0.684 V for 2-NS). 4-WS with wide rib width shows a non-uniform oxygen distribution and a low cell voltage of 0.666 V.

The pressure drop of these flow fields was summarized at 1000 mA cm^−2^ (Table 2). Flow field with smaller channel cross sectional area shows higher pressure drop and larger flow velocity. However, the performance seems not to be consistent with the pressure drop. The pressure drop with 2-NS is triple of that with 1-ND, but these two flow fields show almost the same performance at 0.68 V. Besides, even 4-WS shows a pressure drop two times higher than 2-NS, the voltage with 4-WS is 0.666 V which is ca. 20 mV lower than that with 2-NS. This comparison indicates that the cell performance is more relevant with the under-rib convection than with pressure drop at the current density of 1000 mA cm^−2^, and channel/rib width is the dominant factor for the cell performance.

**Figure 7:**
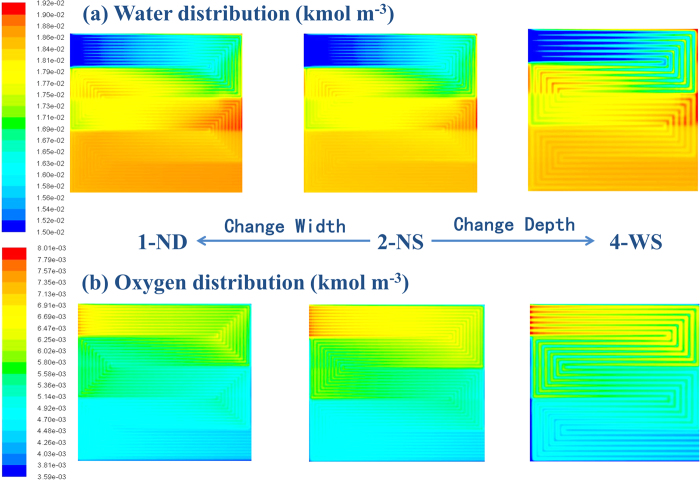
The water and oxygen concentration distribution with 1-ND, 2-NS and 4-WS at 1400 mA cm^−2^ at 100% RH (Cell Temp. = 80 °C and Back Pressure = 150 kPa).

Figure 7 shows the water and oxygen distribution at 1400 mA cm^−2^. At high current density, the flow fields with large pressure drop effectively remove accumulated water in channels, which promotes the oxygen transport. Although the water distribution with 4-WS is still not uniform at high current densities, the large pressure drop prevent an intense flooding issue that happens within 1-ND as shown in Fig. 2(a). This result reveals that the influence of rib width is weakened with the increase of current density and a large pressure drop is needed for water removal. The cell performance with 2-NS becomes better than that with 1-ND at 1400 mA cm^−2^, because both the under-rib convection and the pressure drop are optimized by 2-NS. As shown in Fig. 7(b), the oxygen concentration in channels of 2-NS and 4-WS is much higher than that of 1-ND, confirming the superiority of large pressure drop in water removal.

**Figure 8:**
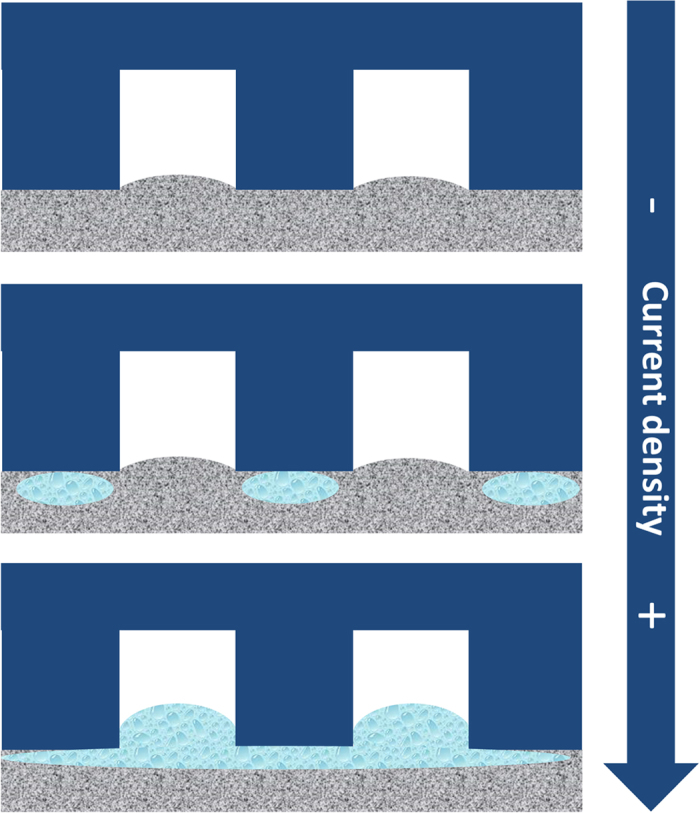
The water accumulation process with the increase of current density.

According to the EIS results and the cell performances at different RH conditions, the difference in cell performance with various flow fields depends on the oxygen transport which is influenced by the accumulated water in both GDL and flow channels. We found that the water distribution directly dominates the cell performance as discussed above. Figure 8 depicts the water accumulation process as the current density increases. From 0 mA cm^−2^ to 500 mA cm^−2^, the current density is rather low that water flooding will not occur. As the current density increases (500 mA cm^−2^ to 1100 mA cm^−2^), water starts to accumulate. Only a small amount of liquid water transports to the channels and it can be easily removed at a low pressure drop; however, the accumulated water in GDL at the under-rib region hinders the oxygen transport to the catalyst layer. Thus, a narrow channel/rib width shows a superior performance by enhancing the under-rib convection. At high current density region (1100 mA cm^−2^ to 1800 mA cm^−2^), a large amount of water accumulates in channels and it requires a flow field with higher pressure drop to purge the accumulated water out.

## Conclusion

We investigated the parallel-serpentine flow fields with different channel geometries, including the channel/rib width and total channel cross-sectional area in this study. The results show that the performance differences with these flow fields derive mainly from transport resistance of oxygen which is determined by the accumulated water and the narrower channels and smaller total channel cross sections would benefit in the cell performance. At low current densities, when water mainly accumulates in GDL, the under-rib convection plays a more important role in water removal than pressure drop does. At high current densities, when a large amount of water accumulates in channels, the pressure drop plays the dominant role in water removal to facilitate the oxygen transport.

## Methods

### Preparation of Membrane Electrode Assembly (MEA)

The MEAs were prepared by decal transfer technique. The ink was prepared by dispersing Pt/C catalysts in DI water with Nafion solution (20 wt. %, DuPont) and isopropanol. Pt (29 wt. %) /VC (TEC10V30E) and Pt (46.7 wt. %) /VC (TEC10V50E) were used for anode and cathode ink respectively. Catalyst ink was sprayed on decal substrate after a ball-milling for 12 h, and dried to form the catalyst layers. The anode and cathode were hot pressed on either side of a Nafion 211 membrane (DuPont) at 145 °C and 1.5 MPa for 3–4 min to fabricate a 25 cm^2^ MEA for the single cell. Nafion ionomer content in the anode and cathode was 20 wt. % and 25 wt. % respectively. Pt loading was 0.05 mg cm^−2^ for anode and 0.4 mg cm^−2^ for cathode.

### Fuel Cell Performance

The performance was tested at 80 °C by an 850e Multi-Range Fuel Cell Test System (Scribner Associates Inc.). Stoichiometric ratio for H_2_: Air and H_2_: O_2_ was 2:2 and 2:9 respectively. These flow field patterns were tested at RH of 100% and 37% with a back pressure of 150 kPa. Electrochemical impedance spectroscopy (EIS) was performed at the cell voltage of 0.6 V and 0.7 V.

### Computational Fluid Dynamics (CFD) Modeling

A three-dimensional, two-phase, isothermal, steady-state model is proposed with following assumptions:

1. The fuel cell operates under steady-state conditions.
2. The reactant gases are incompressible ideal gas.
3. The flow is laminar.
4. The fuel cell works at constant temperature.
5. All the porous media is isotropic.

CFD simulation was resolved by the PEMFC addon module of Fluent (V 6.3.26) at 1000 mA cm^−2^ and 1400 mA cm^−2^ (80 °C, 150 kPa and 100% RH). The mass conservation equations, Navier-Stokes equations and species transport equations were employed for the mass transfer computation. The Butler-Volmer equation was utilized for the electrochemical reaction in the catalyst layer. The geometric models are established by Solidworks, and meshed by ANSYS Meshing. There are 1662828 hexahedral elements in the mesh. The average size of mesh cells was 0.1 mm^3^ and the average mesh quality is 0.82. Grid independency was tested by doubling the number of mesh elements up to about 4 million hex elements. The mass flow rate of each outlet and the pressure & velocity distribution in flow channels are the same with original ones after doubling the mesh number. Therefore, the difference caused by grid can be ignored for further simulation and analysis.

## Additional Information

**How to cite this article**: Wang, C. *et al*. The respective effect of under-rib convection and pressure drop of flow fields on the performance of PEM fuel cells. *Sci. Rep.* **7**, 43447; doi: 10.1038/srep43447 (2017).

**Publisher's note:** Springer Nature remains neutral with regard to jurisdictional claims in published maps and institutional affiliations.

## Figures and Tables

**Table 1:** Details of EIS response data at 0.7 V and 0.6 V at 100% RH (Cell Temp. = 80 °C and Back Pressure = 150 kPa).

	0.7 V			0.6 V		
	i mA cm^−2^	MTR range mOhm cm^2^	MTR width mOhm cm^2^	i mA cm^−2^	MTR range mOhm cm^2^	MTR width mOhm cm^2^
1-ND	864	139–182	43	1422	181–348	167
2-NS	869	148–194	46	1502	139–256	117
3-WD	785	173–238	65	1258	180–342	162
4-WS	785	150–212	62	1331	151–288	137

**Table 2:** Comparison of pressure drop and flow velocity at 1000 mA cm^−2^ and 1400 mA cm^−2^ at 100% RH (Cell Temp. = 80 °C and Back Pressure = 150 kPa).

	1-ND	2-NS	4-WS
@1000 mA cm^−2^			
Cross section (mm^2^)	5.0	2.5	4.2
Voltage(V)	0.681	0.684	0.666
Pressure Drop (Pa)	12.301432	69.440438	67.285556
Velocity _@outlet_ (m/s)	4.819922	12.644443	12.064735
@1400 mA cm^−2^			
Cross section (mm^2^)	5.0	2.5	4.2
Voltage(V)	0.607	0.624	0.584
Pressure Drop (Pa)	20.872942	76.768949	73.148594
Velocity _@outlet_ (m/s)	5.2853999	13.057479	12.637651
